# Supplementary material for: Neuro-inspired optical sensor array for high-accuracy static image recognition and dynamic trace extraction
Source: Nat Commun. 2023 Oct 23;14:6736. doi: 10.1038/s41467-023-42488-9 (PMC10593955; doi:10.1038/s41467-023-42488-9)
Supplement: Supplementary file 1 — Supplementary Information clean [file 41467_2023_42488_MOESM1_ESM.pdf]

Supplementary Information for:

Neuro-inspired optical sensor array for high-accuracy static image recognition and dynamic trace extraction

Pei-Yu Huang[1 #], Bi-Yi Jiang[2, 3#], Hong-Ji Chen[1#], Jia-Yi Xu[2], Kang Wang[4], Cheng-Yi Zhu[1], Xin-Yan Hu[2], Dong Li[1], Liang Zhen[5], Fei-Chi Zhou[2*], Jing-Kai Qin[1*] & Cheng-Yan Xu[1,5*]

[1] Sauvage Laboratory for Smart Materials, School of Materials Science and Engineering, Harbin Institute of Technology (Shenzhen), Shenzhen 518055, China

[2] School of Microelectronics, Southern University of Science and Technology, Shenzhen 518055, China

[3] Department of Applied Physics, The Hong Kong Polytechnic University, Hong Kong 999077, China

[4] Key Laboratory of MEMS of the Ministry of Education, Southeast University, Nanjing 210096, China

[5] MOE Key Laboratory of Micro-Systems and Micro-Structures Manufacturing, Harbin Institute of Technology, Harbin 150080, China

[#] These authors contributed equally to this work.

* Corresponding authors. E-mail addresses: jk.qin@hit.edu.cn; zhoufc@sustech.edu.cn; cy_xu@hit.edu.cn

1. Setup of testing system

The photograph of the home-built testing system is shown in **Supplementary Figure 1**. The NbS_2/MoS_2 optical sensor array is directly integrated into the PCB board for transferring current, and an automatic test system measures the current *via* contact pins around the board. Then, the collected data is processed by customized software and shown on screen in form of a graph.

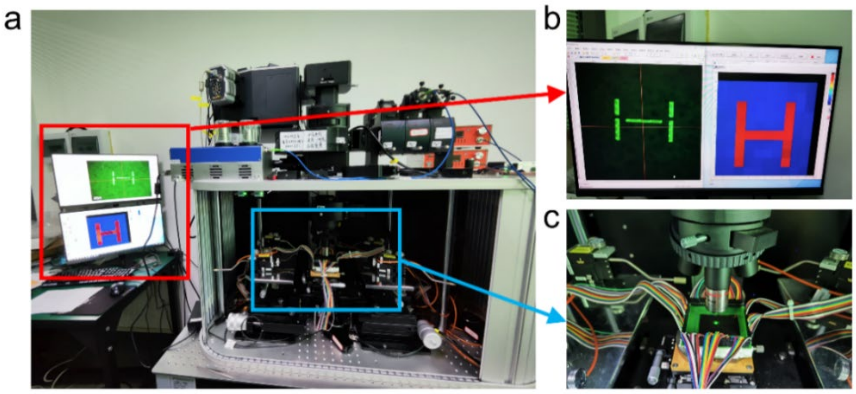

Supplementary Figure 1. Home-built testing system. a Digital photograph of home-built testing system. **b** H shaped photomask and corresponding test result. **c** PCB board with 100 contact pins.

2. Structural, composition and optical properties characterization of NbS_2/MoS_2 heterostructures

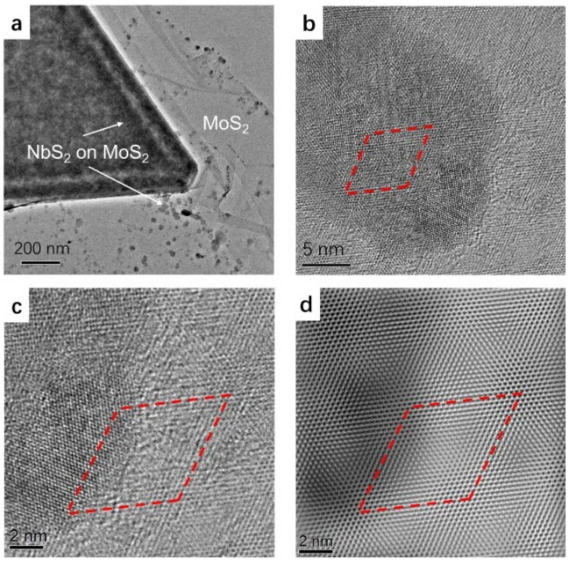

Supplementary Figure 2. Electron microscope characterization of NbS_2/MoS_2 hybrid films. a Low-magnification TEM image. **b-c** HRTEM images and **d** corresponding noise-reduction photograph by IFFT processes. The rhombic selected areas show Moire patterns with the periodicity of 6.5 nm attributed to NbS_2 overlapping upon the MoS_2.

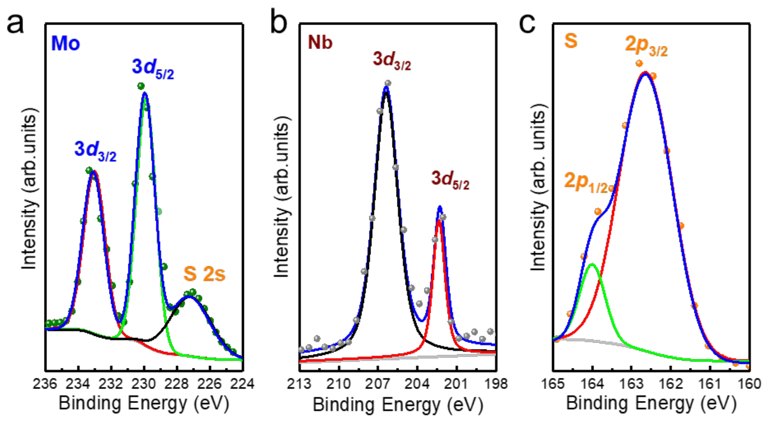

Supplementary Figure 3. XPS spectra of NbS_2/MoS_2 hybrid films. a Mo 3*d*, **b** Nb 3*d*, and **c** S 2*p* spectrum of NbS_2/MoS_2 hybrid films, respectively.

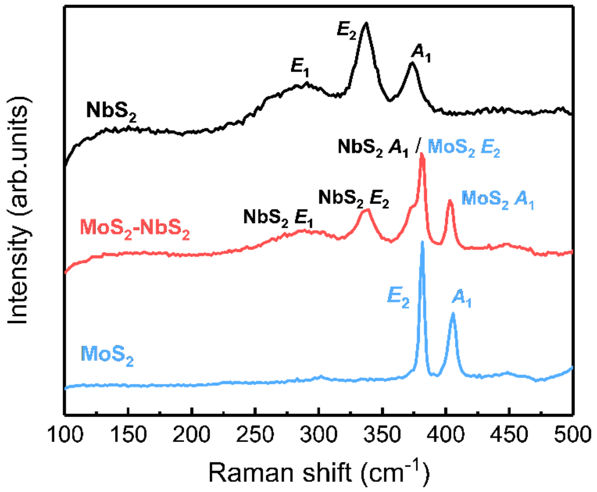

Supplementary Figure 4. Raman spectra of MoS_2, NbS_2, and NbS_2/MoS_2 hybrid films.

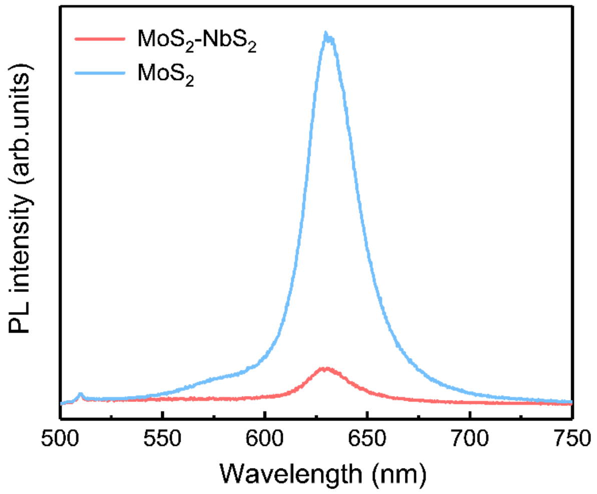

Supplementary Figure 5. PL spectra of MoS_2 and NbS_2/MoS_2 hybrid films.

3. Electrical and optoelectrical measurements of NbS_2/MoS_2 phototransistor

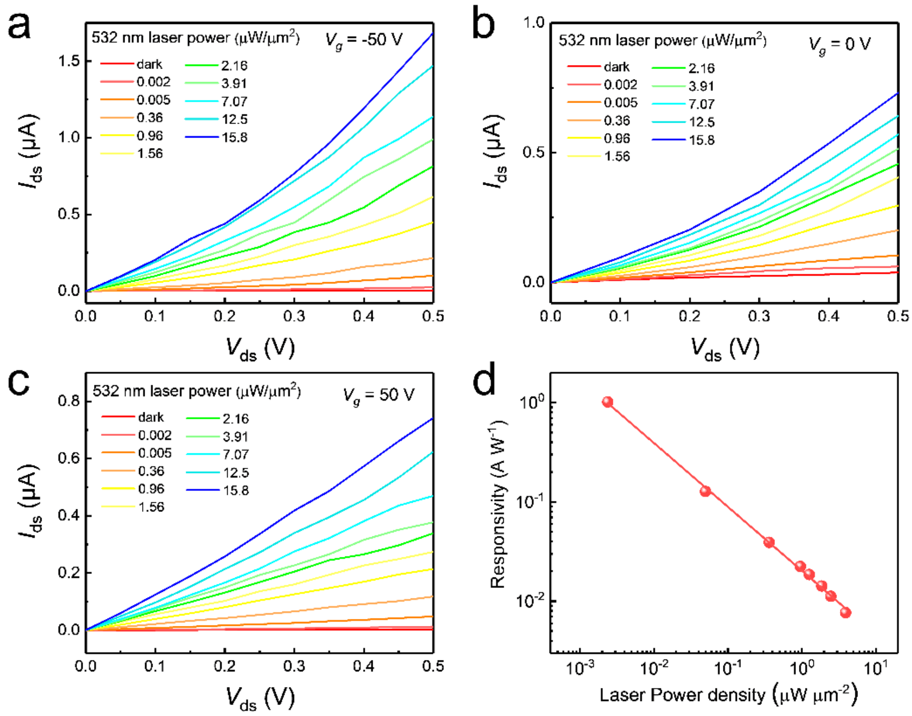

Supplementary Figure 6. Optoelectronic characteristics of NbS_2/MoS_2 phototransistor. Output curves of device with different light intensities at **a** V_{gs}=-50 V, **b** V_{gs}= 0V and **c** V_{gs}=50 V. **d** The light power-dependent responsivity of NbS_2/MoS_2 device.

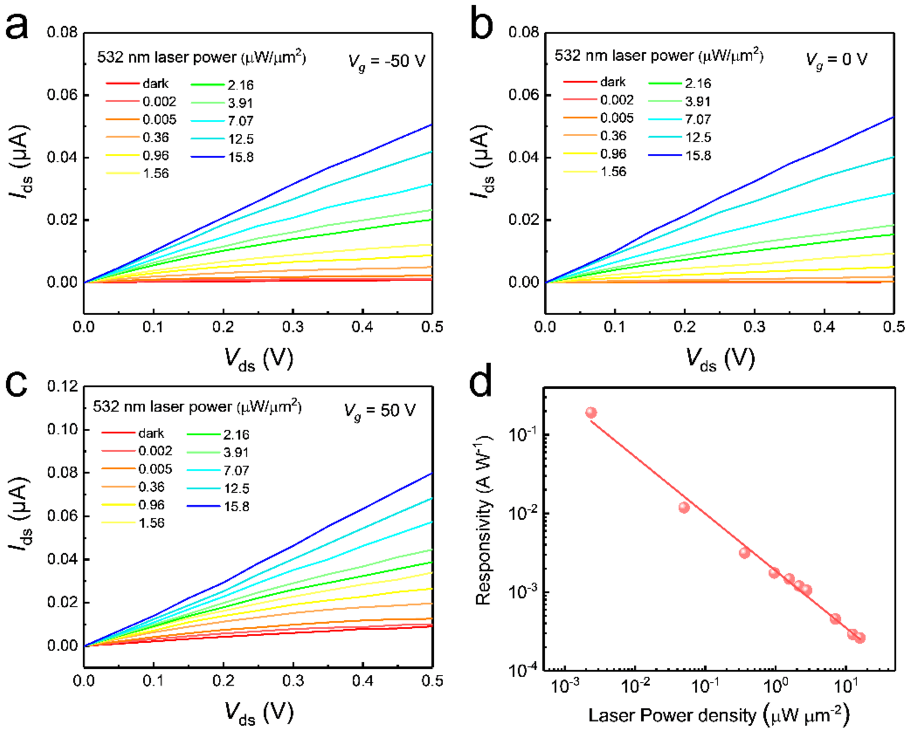

Supplementary Figure 7. Optoelectronic characteristics of MoS_2 phototransistor. Output curves of device with different light intensities at **a** V_{gs}=-50 V, **b** V_{gs}= 0V and **c** V_{gs}=50 V. **d** The light power-dependent responsivity of device.

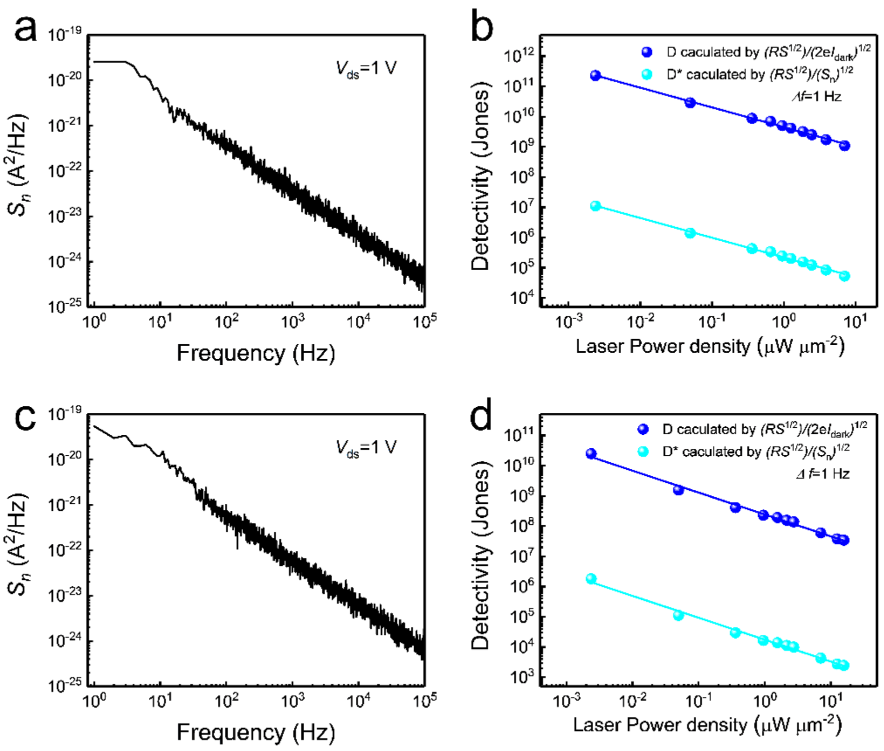

Supplementary Figure 8. Noise density spectrum with respect to frequency (*f*) and real detectivity. a Noise density spectrum of NbS_2/MoS_2 phototransistor, which follows a 1/*f* rule from 1 Hz to 0.1 MHz. **b** Real detectivity (*D**) of NbS_2/MoS_2 phototransistor calculated at 1 Hz. **c-d** Noise density spectrum of MoS_2 phototransistor from 1 Hz to 0.1 MHz and *D** calculated at 1 Hz.

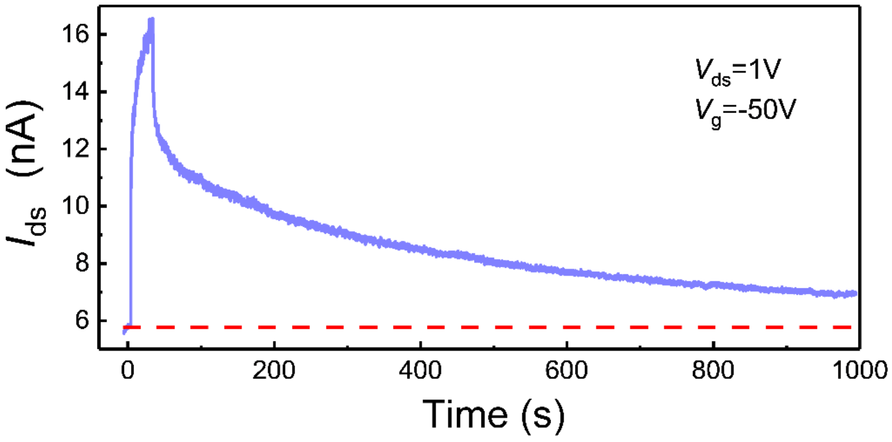

Supplementary Figure 9. Retention characteristics of PPC in NbS_2/MoS_2 optical sensor.

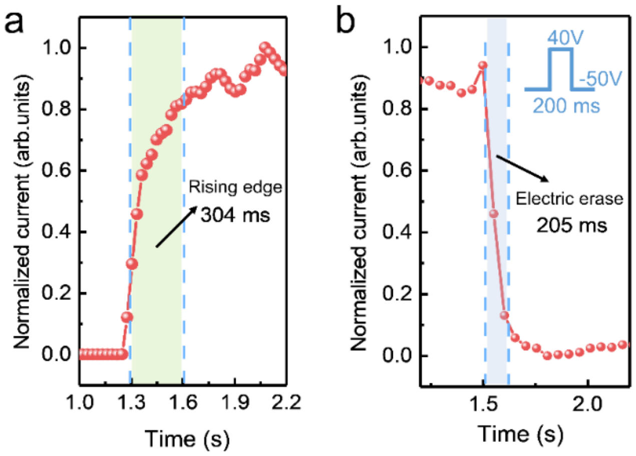

Supplementary Figure 10. Response speed of NbS_2/MoS_2 optical sensor. a Photo-triggered rising time. **b** Electrical reset time, indicating PPC states can rapidly convert to initial stage within 200 ms.

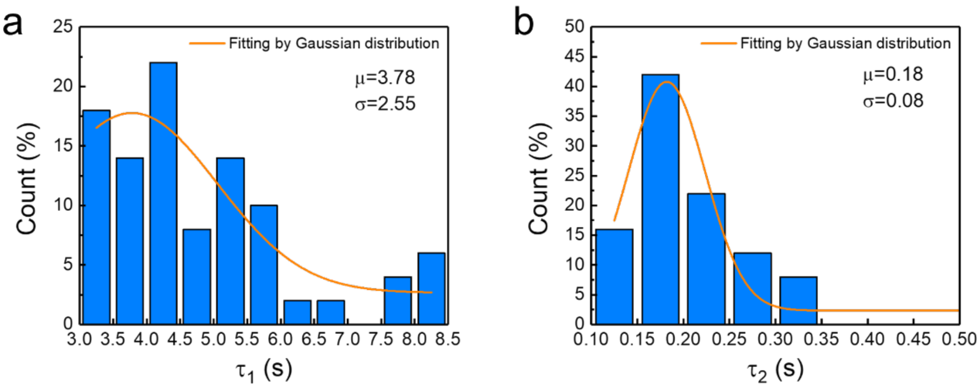

Supplementary Figure 11. Statistic values of characteristic timescales (τ_1 and τ_2) for different pixels. a Slow relaxation times constant (τ_1) statistical distribution with mean values of 3.78 s and corresponding standard deviation of 2.55 s. **b** Rapid relaxation times constant (τ_2) statistical distribution with mean values of 0.18 s and corresponding standard deviation of 0.08 s.

Supplementary Table 1. Comparison of time constants with previous neuro-inspired devices.

Materials	Time constants (τ_1/τ_2)	References
GaO_x	0.28/0.029 s	60
PTHF percentage	0.57/0.072 s	61
P3HT/$CsPbBr_2I$	0.011/0.0003 s	62
$MAPbI_3$/IZO	3.43/0.36 s	63
IGZO/CdS	3.5/0.1 s	64
NbS_2/MoS_2	3.78/0.18 s	Our work

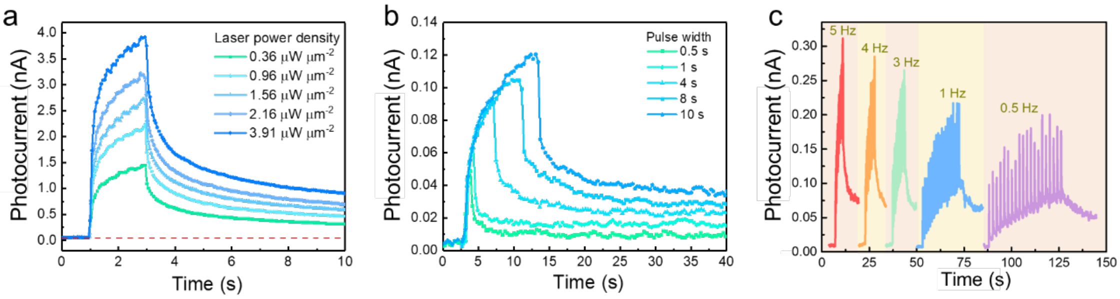

Supplementary Figure 12. Dynamic tunable processes of conductance plasticity for NbS_2/MoS_2 optical sensor. The conductance evolution with the increasing of **a** light intensity, **b** light pulse duration, and **c** frequency of pluses.

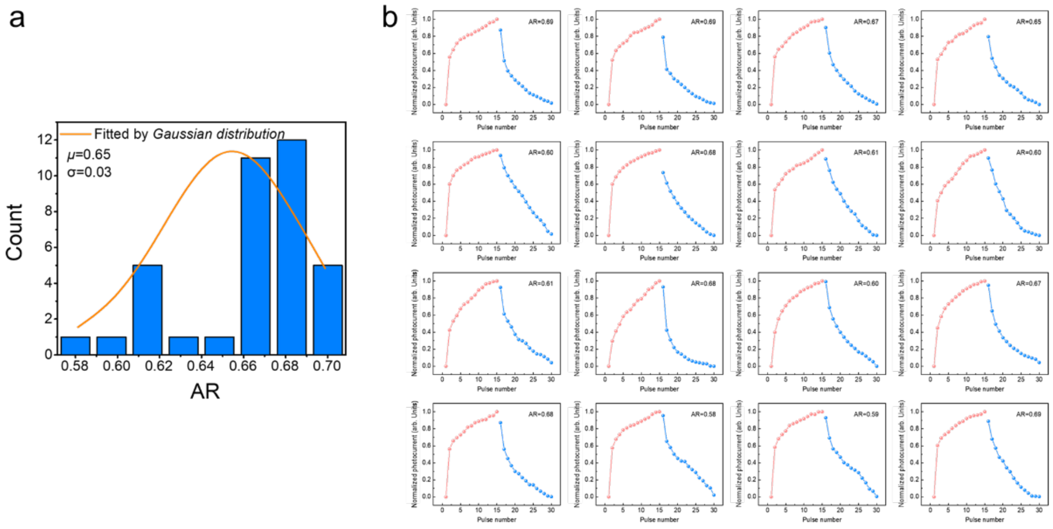

Supplementary Figure 13. AR value distribution for NbS_2/MoS_2 sensor array. a Statistical distribution of AR value is fitted with a logarithmic normal curve, in which the mean values and corresponding standard deviation are 0.65 and 0.03, respectively. **b** AR value of partial NbS_2/MoS_2 devices in sensor array.

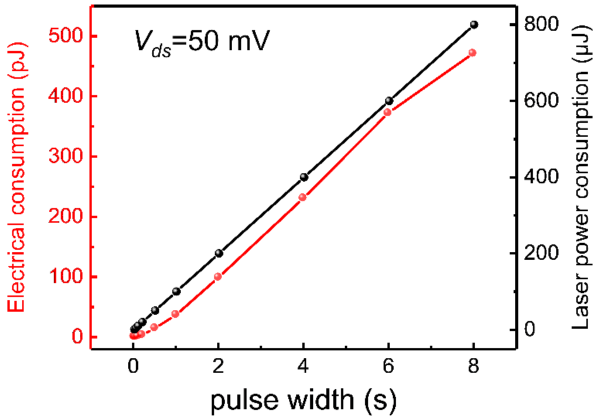

Supplementary Figure 14. The electrical and external light energy consumptions of NbS_2/MoS_2 optical sensor at V_{ds}=50 mV under different light duration. The red spots and line refer to energy consumption of electrical response. The black spots and line refer to energy consumption of external laser illumination.

The ratio of photo-induced persistent photocurrent and initial current is utilized to evaluate device performance. The detailed electrical consumption of NbS_2/MoS_2 optical sensor and current ratio at V_{ds}=50 mV under different light durations is given in **Supplementary Figure 15**.

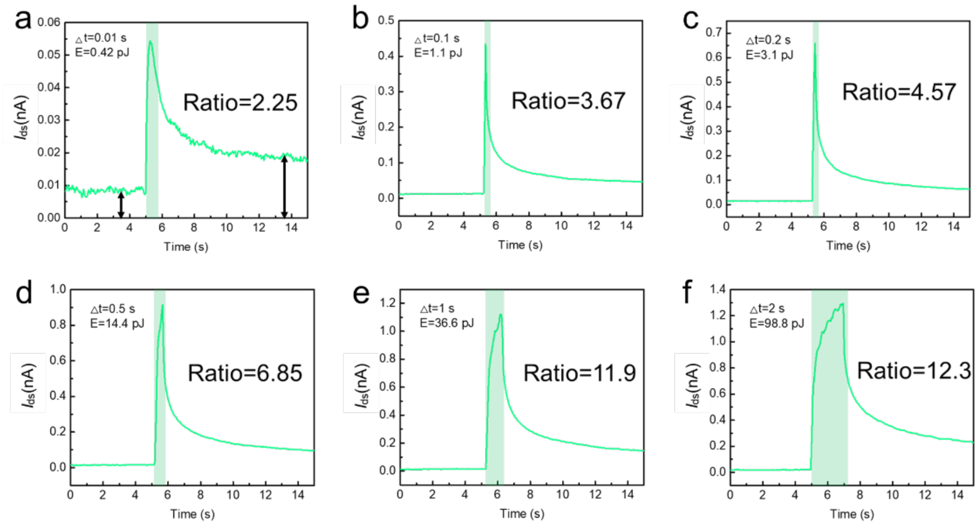

Supplementary Figure 15. Detailed electrical energy consumption and ratio of photo-induced persistent photocurrent and initial current with different pulse duration at V_{ds}=50 mV. a-f Electrical energy consumption and current ratio of NbS_2/MoS_2 optical sensor at V_{ds}=50 mV under light duration from 0.01 s to 2 s.

Table S2. Comparison of electrical energy consumptions for optical neuro-inspired devices

Materials	Wavelength (nm)	Energy consumption	Refs.
MoS_2/PTCDA	532	10 pJ	68
$MoSe_2$/Bi_2Se_3	790	10 nJ	71
In_2O_3/ZnO	UV-Vis.	200 pJ	72
Nb:$SrTiO_3$	Vis.	85 nJ	73
Graphene/SWNTs	405/532	6 nJ	74
ZnO_{1-x}/AlO_y	310	1.7 nJ	75
Black phosphorus	660	924 pJ	76
TiN_xO_{2-x}/MoS_2	365	450 nJ	25
MoS_2/NbS_2	532	0.42 pJ	This work

4. Device-to-device variation of NbS_2/MoS_2 phototransistor

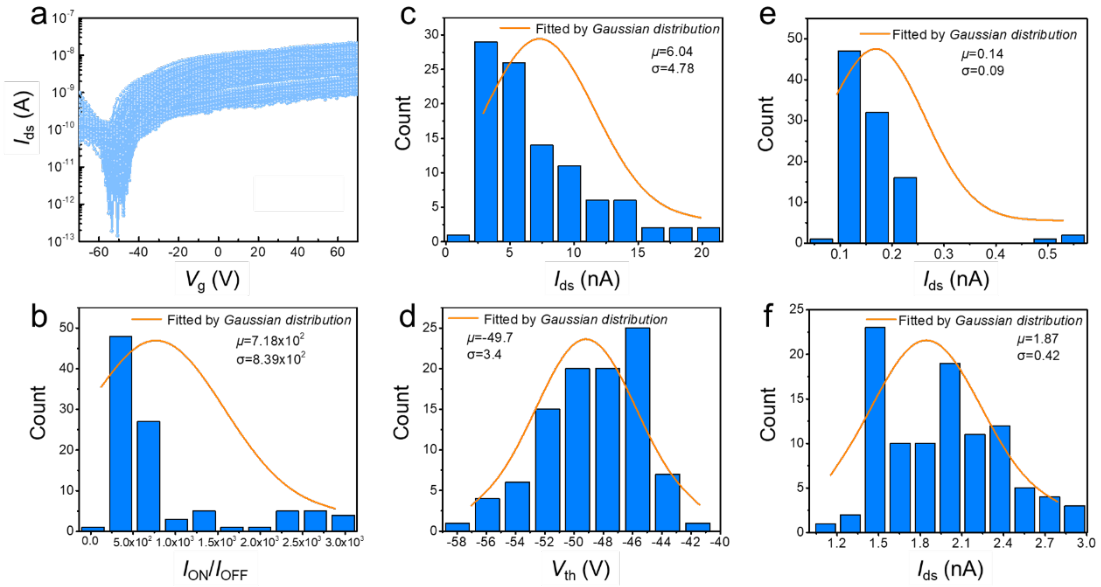

Supplementary Figure 16. Device-to-device variation of NbS_2/MoS_2 optical sensor array. **a** Transfer curves for 100 NbS_2/MoS_2 device. **b** Statistical distribution of ON/OFF ratio. The mean values and corresponding standard deviation are 7.18×10^2 and 8.93×10^2, respectively. **c** Statistical distribution of on-state current (I_{on}). The mean value is 6.04 nA and corresponding standard deviation is 4.78 nA. **d** Statistical distribution of threshold voltage (V_{th}). The mean value is 49.7 V and corresponding standard deviation is 3.4 V. **e** Statistical distribution of dark current (I_{dark}). The mean value is 0.14 nA and corresponding standard deviation is 0.09 nA. **f** Statistical distribution of photocurrent (I_{ph}). The mean value is 1.87 nA and corresponding standard deviation is 0.42 nA.

5. Cross-talk characteristics of NbS_2/MoS_2 phototransistor

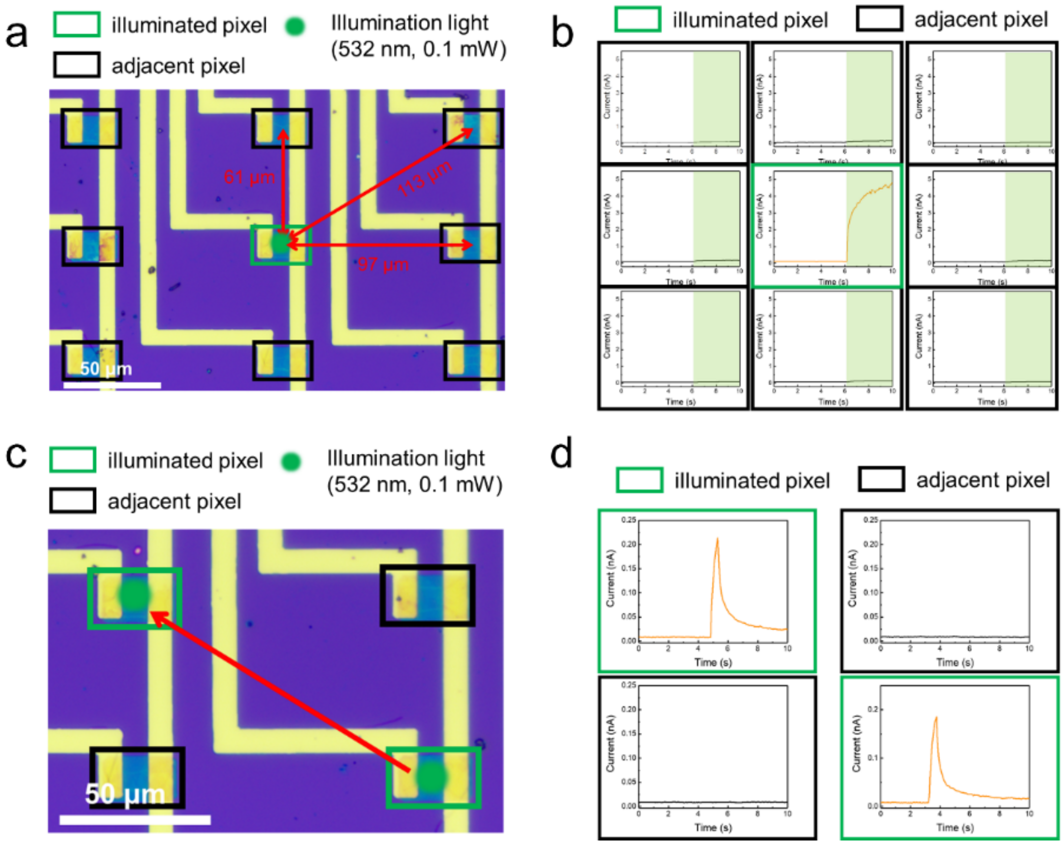

Supplementary Figure 17. Cross-talk characteristics of NbS_2/MoS_2 optical sensor array. **a** OM image of an illuminated pixel and 8 adjacent pixels. The distances between illuminated pixel and adjacent pixels are 97 μm (right, left), 61 μm (top, bottom), and 113 μm (diagonal), respectively. **b** Corresponding photocurrent mapping of illuminated pixel and adjacent pixels. **c** OM image of moving trace and adjacent pixels. The target moves from the bottom right pixel to top left pixel. **d** Corresponding photocurrent mapping of pixels at the trace and outside the trace.

6. Construction of datasets and processing procedure for contrast enhancement

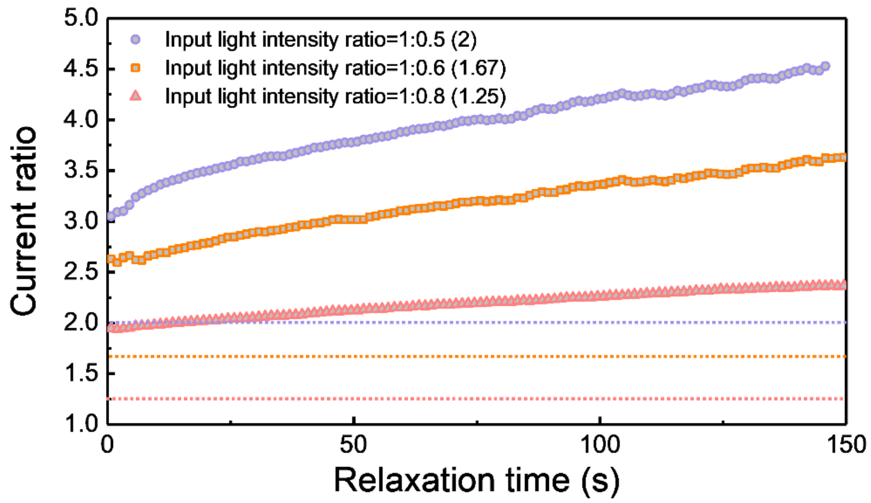

Supplementary Figure 18. Current ratio varied with relaxation time for input light intensity ratio of 1/0.8, 1/0.6 and 1/0.5. The dash lines indicate the original light intensity ratio.

Details on the construction and of datasets and processing procedure for contrast enhancement between target letter and background are introduced as below:

As shown in **Supplementary Figure 19**, handwritten A-J letters images are taken from EMIST dataset as informative letter image, including 24000 training images and 4000 test images. The size of each image is 28×28 pixels.

The unenhanced dataset is created by overlapping a dark background letter and additional random background noise to informative letter images for closing to real scenarios. The light intensity ratio of bright informative letter and background letter is fixed at 1:0.5. The light intensity ratio of bright informative letter and background noise are randomly generated from range 1:0 to 1:0.6. The contrast of images in dataset is defined as the ratio of the average intensity of informative letters to the average intensity of the noise. The noise component is extracted by taking the absolute difference between the noisy image and the expected clean informative letters image. The contrast can be expressed as:

$$\text{Contrast} = \frac{\text{Average(intensity of the informative letter)}}{\text{Average(intensity of the noise)}} \quad (1)$$

Then, enhanced dataset is obtained through Python-simulated contrast enhancement pre-processing of 28×28 NbS_2/MoS_2 sensor array. The whole simulation can be divided into three steps:

(1) The input light intensity for each sensor in the 28×28 array is determined by the gray scale level of the corresponding pixel in the 28×28 sized raw image. The adopted light stimulus is 100 successive light pulses with width of 300 ms. The output current of the simulated 28×28 array is obtained by fitting the relationship between the input light intensity and the output current with actual experimental data. The fitting curve of output current with respect to input light intensity is demonstrated in **Supplementary Figures 20** and **21**.

(2) Device-to-device variance of output current is also considered in simulating array output current. The effect of variance on the array output current is simulated by adding a set of Gaussian noise generated based on the calculated variance. The device-to-device output variance of the array is determined by extracting the average variation of the dark current and photocurrents of the devices under different light intensity from experimental data.

(3) The final simulated current level of the array is obtained by adding the fitted output current and the simulated device-to-device output current variance. The normalized output current mapping of the array is treated as the pre-processed image.

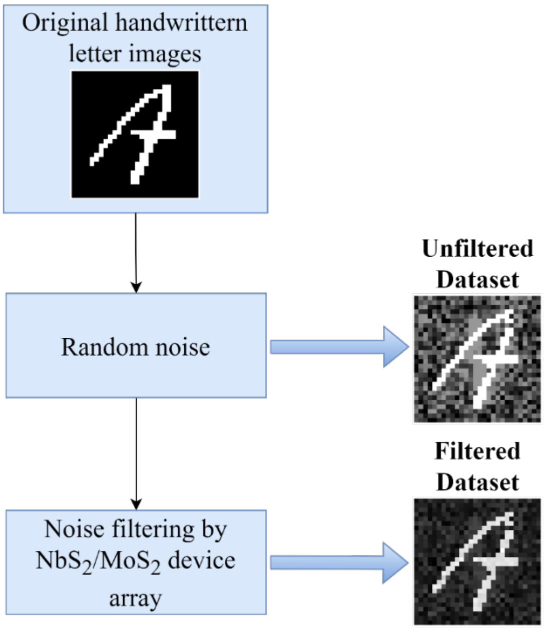

Supplementary Figure 19. Generation of enhanced and unenhanced datasets.

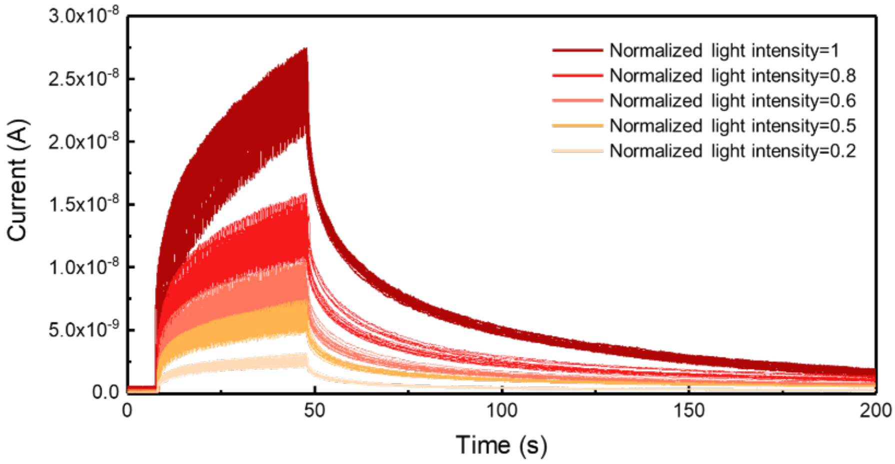

Supplementary Figure 20. Statistics of photocurrent relaxation behavior of devices under different light intensities.

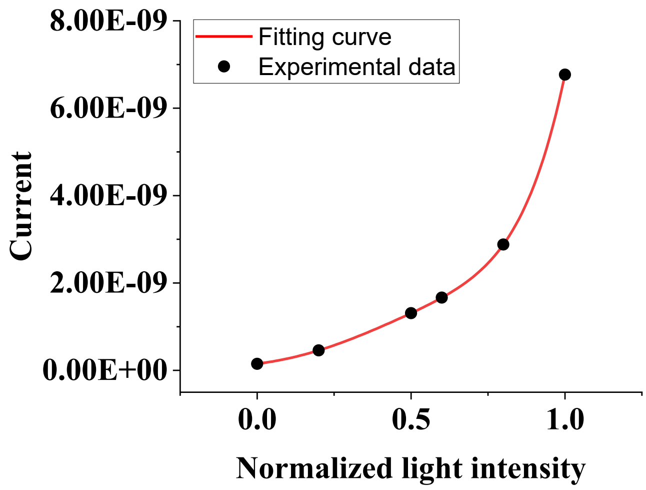

Supplementary Figure 21. Fitting curve of output current with respect to normalized light intensity. The adopted light stimulus for experimental data is 100 successive light pulses with width of 300 ms.

7. Evaluating the effects of array image enhancement pre-processing for image recognition accuracy with both CNN and ANN

To verify the improvement in image recognition accuracy with integrating array image enhancement pre-processing, the enhanced and unenhanced datasets were recognized using two different types of neural networks. As illustrated in **Supplementary Figure 22**, both two datasets were processed using the same artificial neural network (ANN) and the same convolutional neural network (CNN). ANN uses ReLU as the activation function for all fully connected layers except the last layer, which utilizes Softmax function. It is optimized using the Adam function, configurated with beta1 equal to 0.9, beta2 equal to 0.999, and a learning rate of 0.001. The ANN is trained with a batch size of 40 and uses the categorical crossentropy loss function to calculate errors. CNN also employs the same ReLU activation function for all convolution and fully connected layers except the last layer, which adopts Softmax function. CNN is optimized with the identical Adam function parameters as ANN (beta1=0.9, beta2=0.999, learning rate=0.001). The training batch size is also 40. The CNN's convolutional layers are configured with "same" padding to ensure consistent spatial dimensions between input and output feature maps. The categorical crossentropy

loss function is also used in the CNN for error calculation. The results are presented in **Supplementary Figure 23**. From results of both ANN and CNN, we can verify the effectiveness of the array image enhancement function on image recognition.

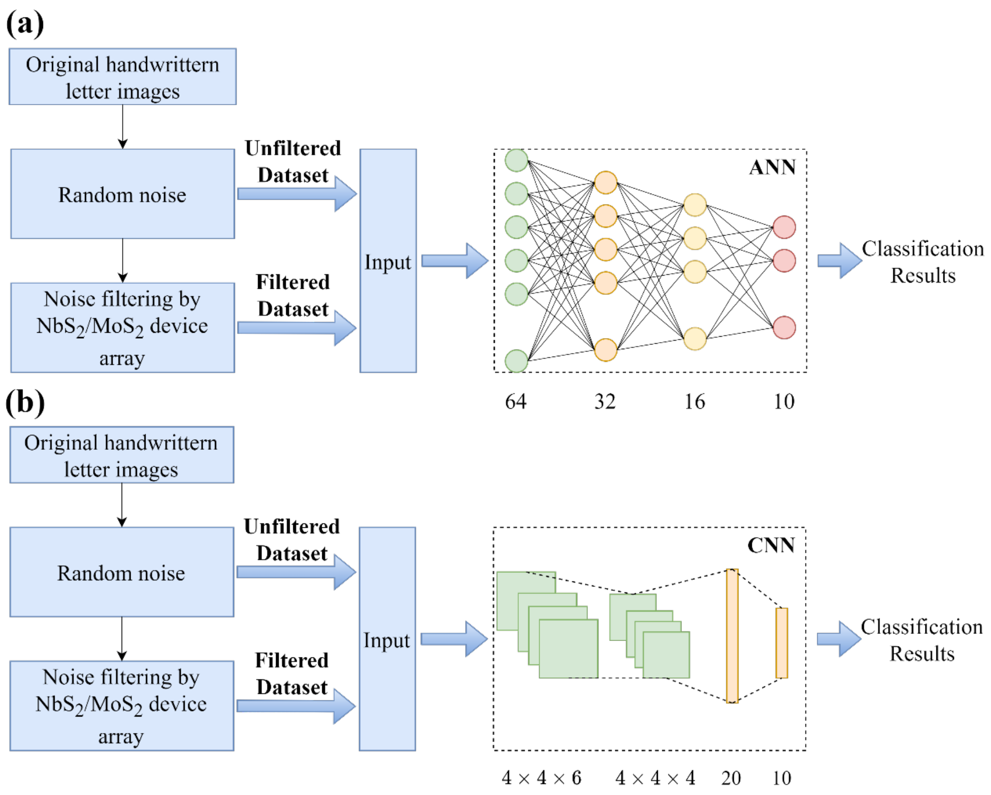

Supplementary Figure 22. Flowchart of the experimental procedure. To verify the image enhancement function of NbS_2/MoS_2 sensor array. Both two datasets were generated and recognized by two types of neural networks: **a** Artificial neural network (ANN) and **b** Convolutional neural network (CNN).

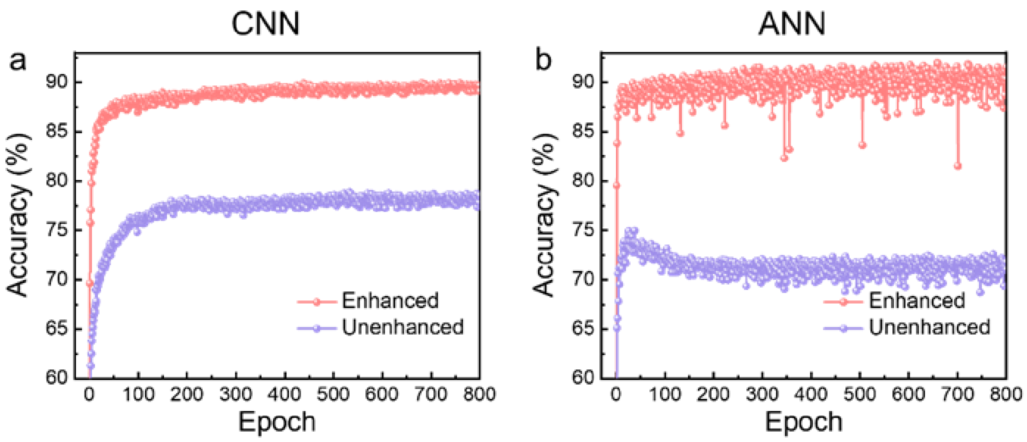

Supplementary Figure 23. Recognition accuracies of two types neural network. a CNN and **b** ANN on two datasets.

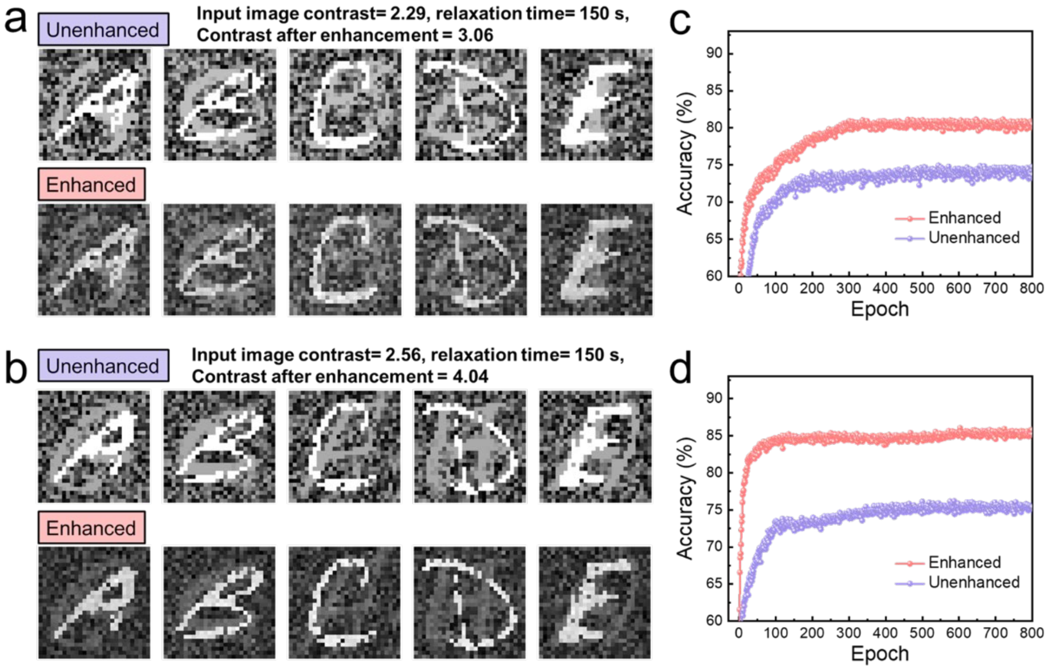

Supplementary Figure 24. Contrast enhancement performances of sensor array and recognition accuracy of CNN for input images with different contrast of 2.29 and 2.56. a-b Comparison of the images before and after pre-processing. **c-d** Recognition accuracies of CNN with and without pre-processing.

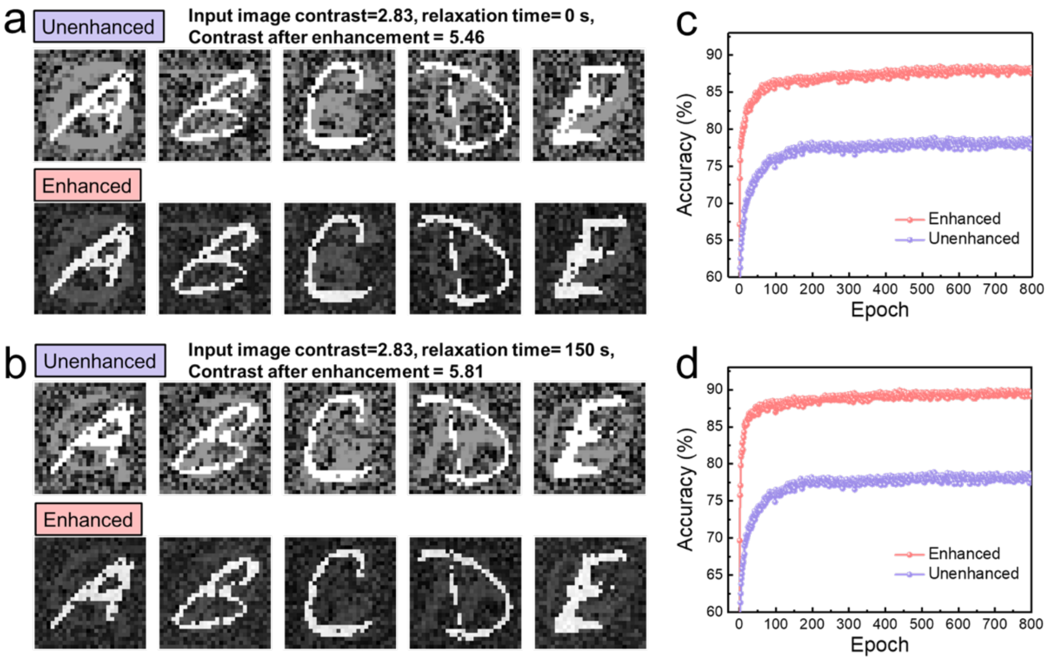

Supplementary Figure 25. Contrast enhancement performances of sensor array and recognition accuracy of CNN for relaxation time of 0 s and 150 s. a-b Comparison of image before and after pre-processing. **c-d** Recognition accuracies of CNN with and without pre-processing.

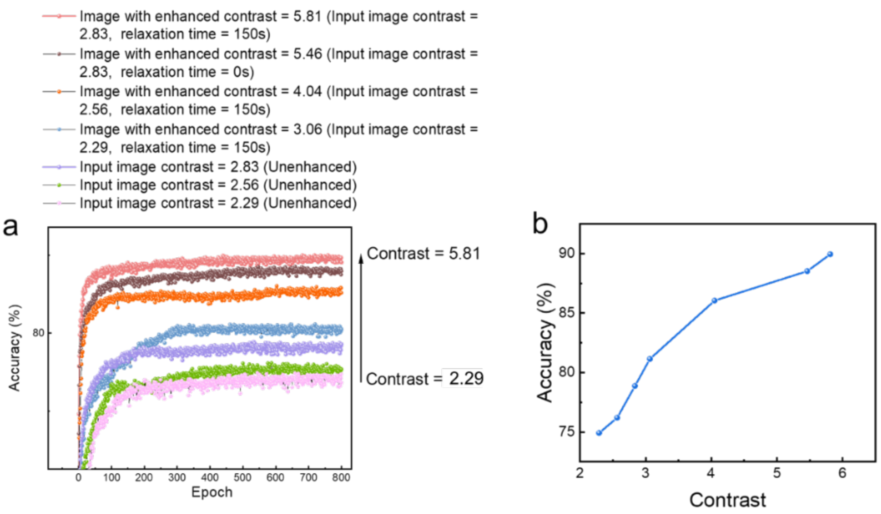

Supplementary Figure 26. Relationship between the contrast value and recognition accuracy of 4 different combinations of laser light intensities for the target and background and different device relaxation times. a Recognition accuracies of the CNNs with 800 training epochs under the 4 different combinations. **b** Relationship between the image contrast value and recognition accuracy.

8. Performance of NbS_2/MoS_2 sensor for trace extraction

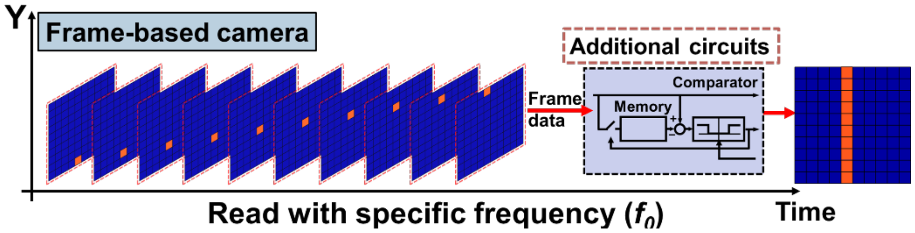

Supplementary Figure 27. Schematic illustration of front-end motion detection realized by frame-based conventional CMOS vision chip.

Supplementary Table 3. Comparison of NbS_2/MoS_2 sensor array with DVS.

	**This work**	Delbruck *et al.* [19]	Boahen *et al.* [21]	Barranco *et al.* [20]
Operation form	**Analog spatial-temporal current**	Event-induced spike signals		
Pixel complexity	**1 phototransistor**	26 transistors, 3 caps, 1 photodiode	38 transistors, 1 photodiode	>15 transistors, 1 photodiode
Sensor area	**1×0.6 mm²**	6×6 mm²	3.5×3.3 mm²	5.5×5.6 mm²
Power consumption	**4.2 nW/Chip 42 pW/Pixel**	24 mW/Chip 1.5 µW/Pixel	62.7 mW/Chip 10.8 µW/Pixel	132 mW/Chip 8 µW/Pixel
Application	**Static and dynamic scenes**	Dynamic scenes		

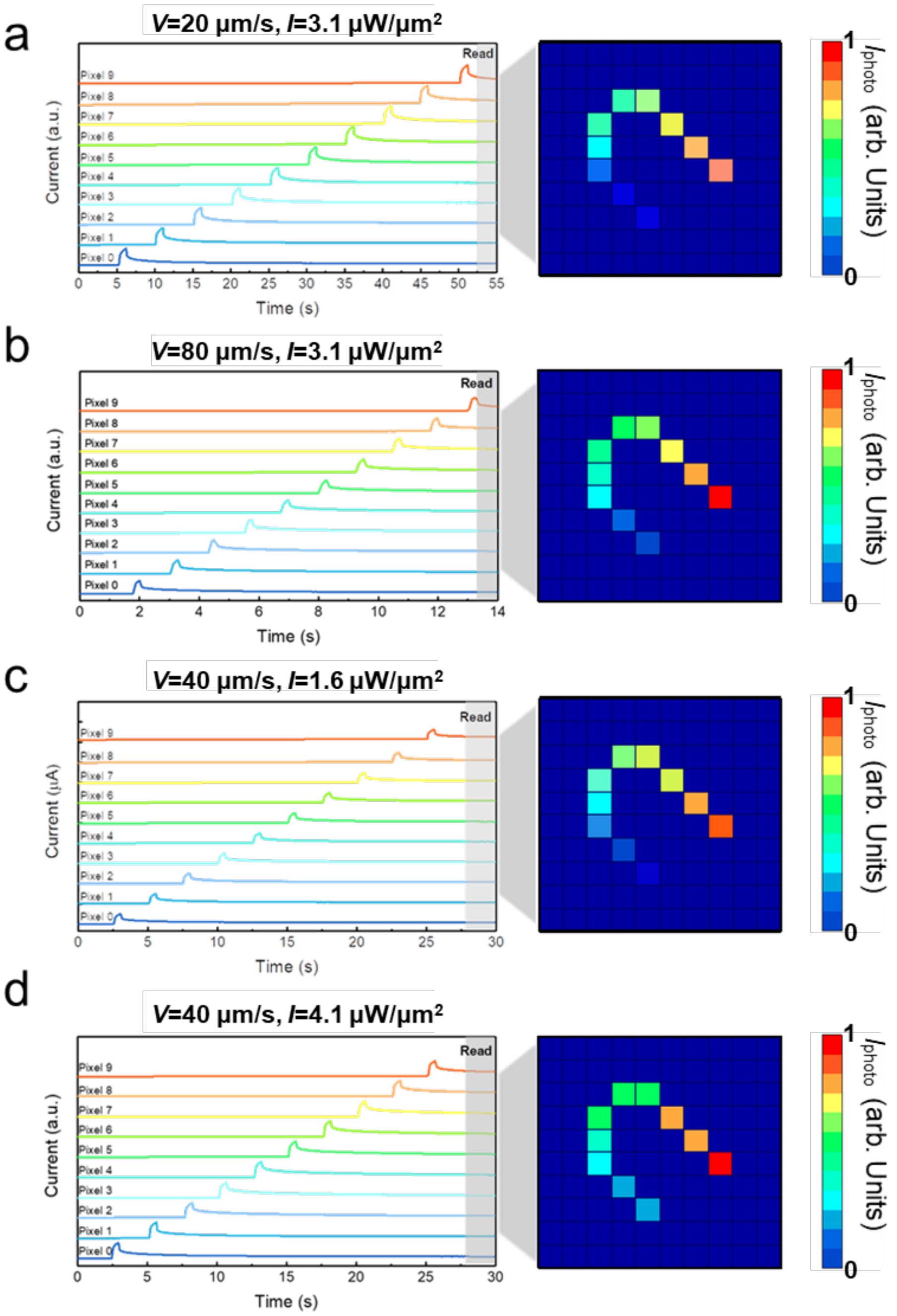

Supplementary Figure 28. Performance of in-sensor trajectory registration for different velocity and light intensity of moving light spot. a-b NbS_2/MoS_2 sensor array for slower and faster trajectory registration. **c-d** NbS_2/MoS_2 sensor array for lower light intensity and higher light intensity trajectory registration (left panel shows real-time photocurrent of pixels located at trajectory, right panel shows current mapping of trajectory registration).

9. Simulation for trajectory registration

The complete workflow of trajectory registration is illustrated in **Supplementary Figure 29**. To generate the trajectory datasets, we developed a random trajectory generating program in the Python programming language that generates trajectories with random starting point, random ending point, and fixed length of 10 on a 10 ×10 background. 10 datasets, each containing 40 unique trajectories, are generated with this random trajectory generating program.

To simulate the output current of the array for the entire duration of each trajectory in the dataset, we first fit the device photocurrent under 3.1 μW μm^{-2} illumination with experimental data in the Python environment (**Supplementary Figure 30**). Then, we used this fitted behavior to simulate the output of each device in the array over the entire duration of each trajectory in the dataset. The output current of the devices in non-trajectory pixels remained at the dark current level, while the output current of the devices in trajectory pixels has the expected decay behavior due to light stimulation. The starting time of the photocurrent decay for each trajectory pixel device was determined through the order in which the devices were passed through. Devices that were passed through earlier have a longer photocurrent decay time.

The photocurrent variance of the devices was also accounted in the simulation. We first extracted the variance of the peak photocurrent and dark current from the decay characteristics of NbS_2/MoS_2 array devices. Then, the measured variance values are utilized as a part of simulation variance for decay process. Gaussian white noise generated from this calculated variance was added into the simulated array output current at every timepoint as another part of simulation variance.

To determine the NbS_2/MoS_2 array trajectory registration accuracy, we restored each trajectory from simulated array output current and compared the registered trajectory to the actual one in trajectory dataset. To determine the order in which the trajectory pixels were passed through in the registered trajectories, we compared the average output photocurrent of each device in the final 5 seconds. The device with larger average output photocurrent was passed earlier. We used the average current over

the final 5 seconds rather than the current at a single timepoint for the purpose of reducing the effect of variance on the light current value and improve the accuracy of trajectory recognition by the array. The array's trajectory registration accuracy on each dataset was calculated as follows:

$$\text{Accuracy} = \frac{\text{Correctly registered trajectory number}}{\text{trajectory number in dataset}} \tag{2}$$

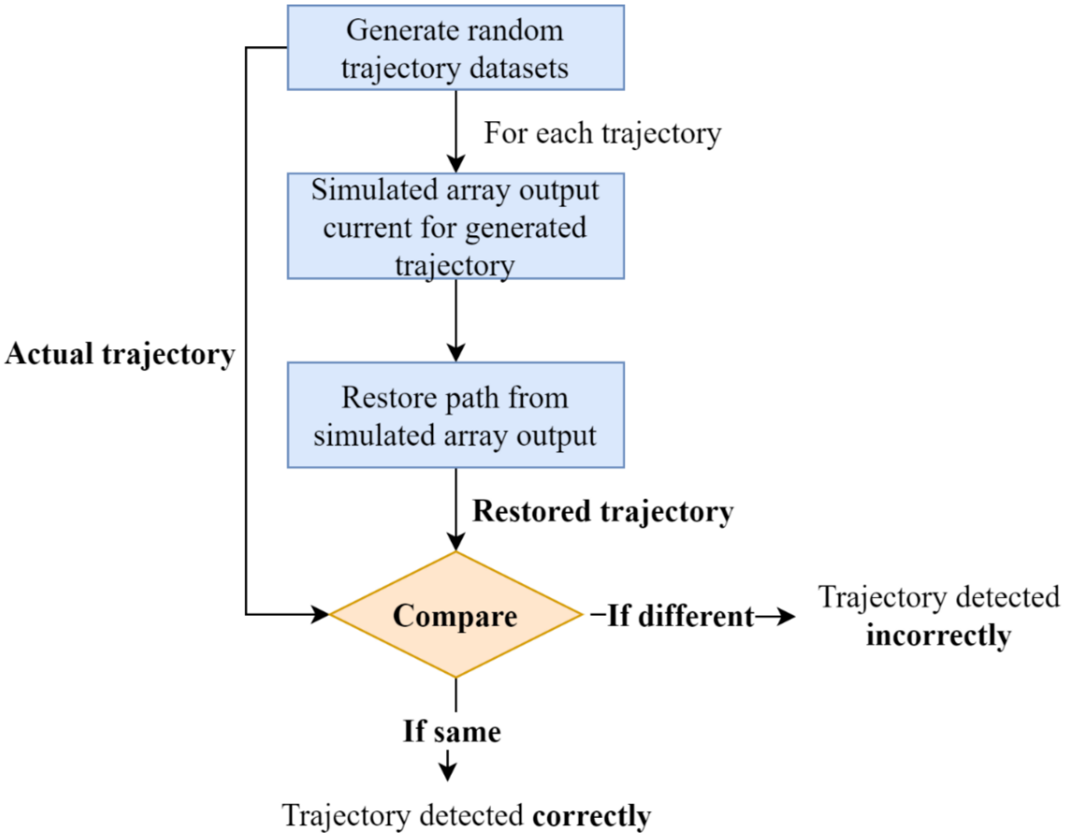

Supplementary Figure 29. Workflow of the trajectory registration.

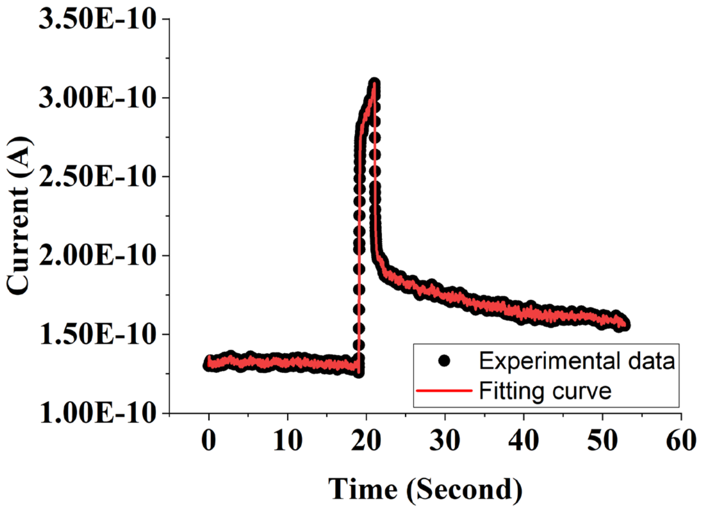

Supplementary Figure 30. Fitted curve of NbS_2/MoS_2 device current under 3.1 μW μm^{-2} light illumination.

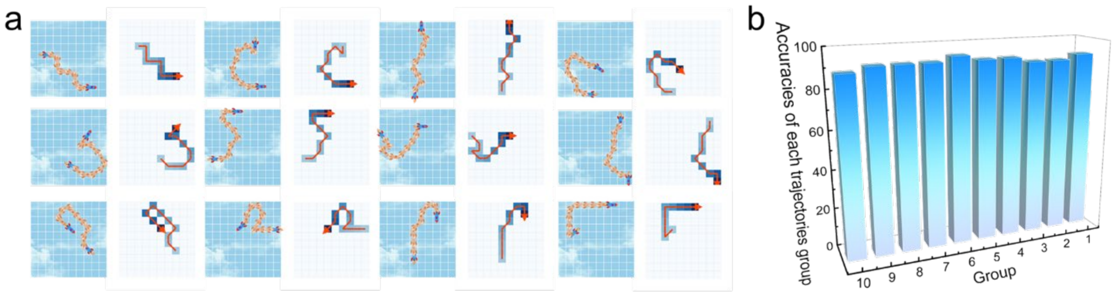

Supplementary Figure 31. The simulation result of trajectory registration. a 12 typical registered and corresponding input traces. The starting point, ending point, and moving direction of each trajectory are all random, ensuring that a large population of possible trajectories can be detected by NbS_2/MoS_2 sensor array. **b** Registration accuracies of 10 trajectory groups.

10. Fabrication process for NbS_2/MoS_2 sensor array

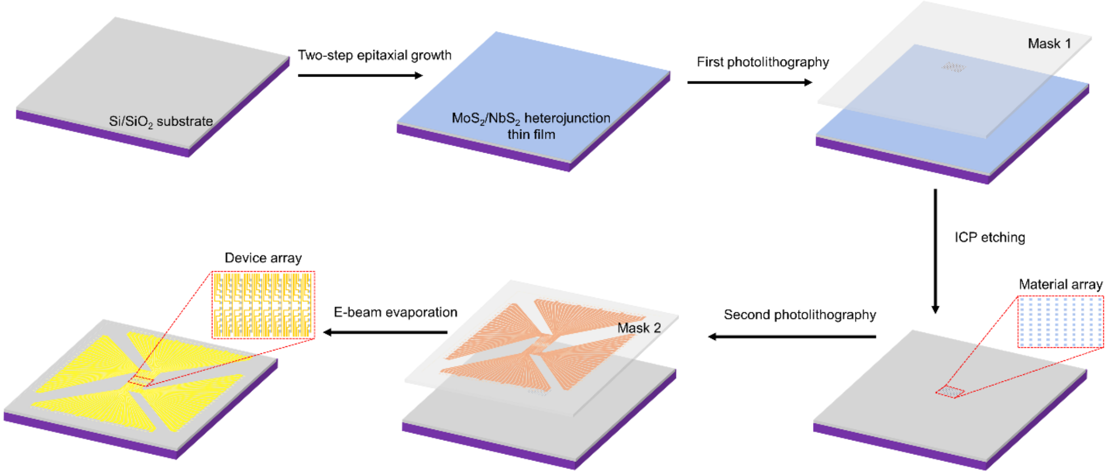

Supplementary Figure 32. Schematic diagram of the procedures for fabricating NbS_2/MoS_2 sensor array.
